# Surgical care of thoracic malignancies during the COVID‐19 pandemic in México: An expert consensus guideline from the Sociedad Mexicana de Oncología (SMeO) and the Sociedad Mexicana de Cirujanos Torácicos Generales (SMCTG)

**DOI:** 10.1111/1759-7714.13546

**Published:** 2020-07-06

**Authors:** Jose Corona‐Cruz, Enrique Guzmán‐de Alba, Marco Iñiguez‐García, Raúl López‐Saucedo, Carlos Olivares‐Torres, Jeronimo Rodriguez‐Cid, Gustavo Salazar‐Otaola, Héctor Martínez‐Said, Raja M. Flores, Oscar Arrieta

**Affiliations:** ^1^ Thoracic Surgical Oncology Instituto Nacional de Cancerologia Mexico City Mexico; ^2^ Thoracic Surgery Centro Médico ABC Mexico City Mexico; ^3^ Thoracic Surgery Instituto Nacional de Enfermedades Respiratorias Mexico City Mexico; ^4^ Surgery Division Centenario Hospital Miguel Hidalgo Aguascalientes Mexico; ^5^ Thoracic Surgery Hospital General de Tijuana Tijuana Mexico; ^6^ Thoracic Oncology Instituto Nacional de Enfermedades Respiratorias Mexico City Mexico; ^7^ Surgical Oncology Instituto Nacional de Cancerologia Mexico City Mexico; ^8^ Division of Thoracic Surgery Mount Sinai School of Medicine New York New York USA; ^9^ Thoracic Oncology Unit Instituto Nacional de Cancerologia Mexico City Mexico

**Keywords:** COVID‐19, lung cancer, surgical oncology, thoracic oncology, thoracic surgery

## Abstract

To date, the impact, timeline and duration of COVID‐19 pandemic remains unknown and more than ever it is necessary to provide safe pathways for cancer patients. Multiple triage systems for nonemergent surgical procedures have been published, but potentially curative cancer procedures are essential surgery rather than elective surgery. In the present and future scenario of our country, thoracic oncology teams may have the difficult decision of weighing the utility of surgical intervention against the risk for inadvertent COVID‐19 exposure for patients and medical staff. In consequence, traditional pathways of surgical care must be adjusted to reduce the risk of infection and the use of resources. It is recommended that all thoracic cancer patients should be offered treatment according to the accepted standard of care until shortage of services require a progressive reduction in surgical cases. Here, we present a consensus of recommendations discussed by a multidisciplinary panel of experts on thoracic oncology and based on the best available evidence, and hope it will provide a modifiable framework of guidance for local strategy planners in thoracic cancer care services in Mexico.

**Key points:**

**Significant findings of the study:**

This article provides recommendations to guarantee the continuity of surgical care for thoracic oncology cases during COVID‐19 pandemic, whilst maintaining the safety of patients and medical staff.

**What this study adds:**

This guideline is the result of an expert consensus on thoracic surgical oncology with recommendations adapted to medical, economic and social realities of Mexico.

## Abstract 2

Consensus statement to guide the practice of thoracic surgical oncology during COVID‐19 pandemic in México.

## Introduction

Currently, several organizations have published recommendations for cancer care during COVID‐19 pandemic.^1^, ^2^ Multiple triage systems for nonemergent surgical procedures have also been published;^3^, ^4^ however, local publications from Mexican experts and adapted to our reality are scarce.^5^

In surgical oncology it is important to distinguish that potentially curative cancer procedures are “essential surgery” rather than “elective surgery”.^3^ For a few cancer patients, surgery may be delayed for months without negative consequences; however, for most thoracic cancer cases, surgery is the only opportunity for cure and a failure to perform an indicated surgery in timely fashion can be detrimental for their survival.^6^

We must keep in mind that cancer surgery requires a lot of health care resources including operating rooms (OR), intensive care units (ICU), ventilators, blood products, personal protection equipment (PPE) and basic medical supplies. In addition, every surgical patient requires many human resources (ie, surgeons, residents, anesthesiologists, and nurses, among others). During this pandemic, the limitation in access to diagnostic testing, shortage of protective supplies for providers and limited hospital capacity (that includes ICU beds) may result in a conflict with care delivery for those patients with COVID‐19.^7^

Thoracic surgery has two associated risks in this pandemic: impairment of lung function in patients and potential exposure of the surgical team to aerosolized COVID‐19 particles. With this in mind, thoracic oncology teams may face a difficult decision of weighing up the utility of surgical intervention against the risk of inadvertent COVID‐19 exposure to patients and medical staff.^8^ In consequence, traditional pathways of surgical care must be adjusted to reduce the risk of infection and use of resources.

The following recommendations were discussed in a multidisciplinary teleconference and are based on the best available evidence. We hope they can provide a modifiable framework of guidance for local strategy planners in thoracic cancer care services in México to guarantee the continuity of treatment for their patients. We suggest that exact workflows be center‐specific depending on its particular situation taking into account the local status of the pandemic and the local availability of resources.

## General measures in the preoperative setting

A patient that engages with the traditional surgical care system will require clinic visits, laboratory and imaging tests; also, inpatient stay results in a considerable number of personal contact points and many potential opportunities for viral transmission.^7^ We know that patients with lung cancer and other thoracic malignancies are considered at high risk of mortality with COVID‐19 and are especially vulnerable to adverse outcomes after lung surgery.^9^, ^10^

The following recommendations are aimed to minimize the need for hospital attendance and stay, whilst maintaining an appropriate and effective surgical pathway:

- Preoperative full lung function testing is not necessary when the clinician and the surgeon are comfortable with spirometry results.^11^
- Surgery must be planned to minimize the length of stay; minimally invasive access has shown equivalent oncologic results in most thoracic malignancies and should be considered as the first option whenever possible.^12^
- For malignant pleural effusion (MPE), indwelling pleural catheter (IPC) insertion must take preference over procedures that require inpatient stay (ie, pleurodesis). Also, consider the need for further pleural interventions when planning only a pleural aspiration. It is recommended that family members are trained in the drainage of IPCs at home.^13^
- For diagnostic purposes, outpatient percutaneous image‐guided biopsy would be preferrable over surgery.^12^
- When possible, plan multiple procedures for a single hospital visit (ie, biopsy, pleural interventions).^11^
- In most countries, a nationwide blood products shortage will result from social distancing practices, and blood stocks must be preserved for emergency surgeries, active bleeding and a few nonurgent cancer surgeries.^7^
- Telemedicine has been suggested as an alternative to clinic visits in many guides, and we encourage its use.^14^ However, we must take in account that in‐hospital infrastructure for this practice is variable in Mexico and high‐speed internet access is limited in most parts of our country.^15^

## General measures in the perioperative setting

Much has been learned from COVID‐19 in the last months, but a lot remains unknown. The most important question is whether routine surgical procedures can be done safely, as protecting OR staff should be a priority. COVID‐19 is mostly a respiratory disease, and the main transmission is through droplets and aerosolization, and therefore a policy of universal respiratory precautions in the OR is encouraged^16^ and includes the following key points:

- Implement a triage to identify elective patients with symptoms of an acute COVID‐19 infection and those with a previously documented infection.^14^
- In‐hospital isolation and testing for SARS‐CoV‐2 in the previous 48 hours is strongly recommended for all elective cases.^16^
- If a nonurgent patient has evidence of COVID‐19 infection, surgery should be deferred at least 14 days and until the repeated test is negative for COVID‐19 infection.^14^, ^16^
- As sensitivity of current tests ranges from 31% to 60%,^17^ all patients who are not tested or test negative should be assumed to be potentially infected with SARS‐CoV‐2.
- Assuming that every patient entering the OR has the virus, an adequate supply of personal protective equipment (PPE) to all OR staff is encouraged (addition of N95 respirators and eye protection to standard OR protocols).^14^, ^16^
- Procedures that generate fine aerosols represent a higher risk of infection and OR personnel should take measures to avoid exposure to aerosolized virus during surgery (examples: electrocautery, insufflation gases, opening of lung parenchyma or airway).^16^
- Chest tubes and pleural drainages constitute aerosol‐generating devices and concern has been raised especially in those patients with postoperative air leaks.^18^ To our knowledge. there is no published data regarding the specific risk of SARS‐CoV‐2 transmission via this route; however, we recommend the modification of chest tube drainages to minimize the potential aerosolization of the virus. Most used modifications include adding dilute bleach to water chamber and the attachment of a filter to the suction port. ^19^ Specific directions for modifications can be found in the articles referenced here. ^18^, ^19^

## Triage for bronchoscopy procedures in cancer patients

Bronchoscopy refers to all flexible, rigid, interventional and endobronchial ultrasound procedures. It must be acknowledged that all bronchoscopy procedures are aerosol‐generating procedures (AGPs) and therefore indications for these procedures should take into account the high risk of COVID‐19 transmission.^20^

- Bronchoscopy for patients with nonmalignant (or preinvasive) conditions could be postponed without significant risk for the patient.^12^, ^20^
- In malignant cases it is recommended to avoid these procedures as a diagnostic and staging tool as much as possible. Alternatives as image‐guided percutaneous biopsy or noninvasive staging with PET‐CT scan should be considered^12^, ^20^
- If there are no available alternatives to bronchoscopy, or it is considered as mandatory, experts suggest testing for COVID‐19 infection prior to performing the procedure. If the result is negative, bronchoscopy could proceed, but if the result is positive, the procedure should be deferred for at least 14 days and until the repeated test is negative for COVID‐19 infection^20^, ^21^

## Triage for nonurgent thoracic surgical oncology cases

To date, the impact, timeline and duration of pandemic remains unknown and more than ever it is necessary to provide safe pathways to guarantee treatment for cancer patients. Although reorganization of hospitals and medical facilities to provide COVID‐19 free hospitals to avoid treatment interruptions have been encouraged by many experts,^22^ we acknowledge that feasibility of this strategy will be variable across our country.

We recommend that all thoracic cancer patients should be offered treatment according to the accepted standard of care until, and only if, shortage of services require a progressive reduction in surgical cases. Patients who are most likely to be harmed by a delay, or a change to a nonsurgical treatment, should be then prioritized; and those whose treatment is delayed or deferred should be tracked to avoid an interruption of cancer care.^23^

A “priority” classification has been suggested to categorize cases based on severity of an individual patient's condition and potential efficacy of treatments.^24^

### Priority A category

Priority A patients have conditions that are immediately life‐threatening, clinically unstable, or complete intolerable and for whom even a short delay would significantly alter the patient's prognosis. These cases should be given top priority even if resources become scarce.

### Priority B category

Priority B cases includes those who do not have immediately life‐threatening conditions, but for whom treatment should not be indefinitely delayed until the end of the pandemic. If local conditions allow for surgery only for priority A cases, priority B cases can be delayed for a defined period of time no longer than 12 weeks, or should be allocated to a nonsurgical modality of treatment.

### Priority C category

Patients in this category are those for whom surgery can be indefinitely deferred until the pandemic is over, or those in whom a nonsurgical approach should be considered as first option.

A list of our recommendations and the suggested treatment alternatives are summarized at Table 1.

**Table 1:** Summary of triage recommendations for surgical care for thoracic malignancies during COVID19 pandemics

Priority	Patient description	COVID‐19 recommendations
**A**	**Urgent surgery** Tumor hemorrhage not amenable for nonsurgical treatment^11^, ^25^ Perforated tumor^11^, ^25^ Tumor associated infection^11^, ^25^ Management of surgical complications (ie, hemothorax, empyema)^11^, ^25^ Threatened airway^11^, ^25^ Symptomatic malignant pleural effusion^13^ Symptomatic malignant pericardial effusion^13^	In all cases, proceed as soon as possible
**A**	**Essential surgery** Solid or predominantly solid (>50%) lung nodule^26^ Stage I and II NSCLC^11^, ^12^, ^25^ Post induction therapy lung cancer^11^, ^25^ Symptomatic mediastinal tumors (excluding lymphomas, germ‐cell tumors and thymic carcinomas amenable to chemotherapy)^11^, ^25^ Chest wall tumors with high malignant potential (ie, sarcomas)^11^, ^25^ Patients enrolled in therapeutic clinical trials^11^, ^12^, ^25^ VATS for staging or diagnostic indispensable to start treatment^11^, ^25^ Moderate to large malignant pericardial effusion in asymptomatic patient^13^ Residual mass in germ cell tumors^11^, ^25^ Symptomatic benign tumors^11^, ^25^	In all cases, proceed as soon as possible
**B**	**Cases that could be deferred**	
	NSCLC <2 cm without nodal disease^23^, ^25^, ^26^	Consider SABR if available and surgical capacity is reduced
	NSCLC after radical chemo/radiation^27^	Consider surveillance or immune therapy with durvalumab if available
	Asymptomatic (nonbulky) thymomas^11^, ^25^	Reschedule as soon as possible
	Asymptomatic (nonbulky) carcinoid tumors^11^, ^25^	Reschedule as soon as possible
	Asymptomatic (nonbulky) posterior mediastinal tumors^11^, ^25^	Reschedule as soon as possible
	Pulmonary metastases (unless surgery will impact subsequent treatment or no better alternative of treatment available)^11^, ^25^	Consider nonsurgical treatment alternatives
	High surgical risk patients (ie, not fit for a lobectomy, requires pneumonectomy, need for ICU)^11^, ^25^, ^27^	Consider nonsurgical treatment alternatives
	VATS procedures in asymptomatic patients with suspected mesothelioma^11^, ^13^, ^25^	Consider image‐guided procedures
	Asymptomatic benign tumors^11^, ^25^	Reschedule as soon as possible
**C**	**Alternative treatment strongly recommended**	
	Suspicious lesions that includes predominantly ground‐glass or part solid (<50%) lung nodules^26^	CT surveillance it is accepted
	Stage III NSCLC^27^	Radical chemo/radiation is strongly recommended
	Oligo‐metastatic stage IV NSCLC^11^, ^12^, ^25^	Consider nonsurgical procedures
	Mesothelioma surgery^25^, ^26^	Prefer nonsurgical approaches only; if surgery is considered, P/D should be the first option
	Diagnostic bronchoscopy^18^, ^19^	Prefer image guided biopsy
	Staging bronchoscopy^18^, ^19^	Prefer noninvasive staging such as PET‐CT scan or mediastinoscopy/VATS
	Stenting or debulking procedures^11^, ^25^	In a palliative setting, risk for patient and surgical staff is higher than the benefit in most cases

NSCLC, non‐small cell lung cancer; VATS, video‐assisted thoracoscopic surgery; SABR, stereotactic ablative body radiation; CT, computed tomography; ICU, intensive care unit; PET‐CT, positron‐emission tomography ‐ computed tomography; P/D, pleurectomy/decortication.

### Conclusions

Our recommendations cannot cover all clinical scenarios. Surgeons must use their experience and judgment regarding which patients need to proceed with a nonurgent surgery versus those who can tolerate a delay, or those who would be better served with a nonsurgical approach. Multidisciplinary teams will always make the final decision on the most appropriate action for individual patients and their local services.

All decisions, including the risk of proceeding or deferring an operation, must be discussed with the patient. Ideally, institutional policies must be implemented so the risk will be balanced carefully, and resources will be utilized wisely.

A strong leadership from surgeons and rapid communications are needed to ensure rapid implementation of protocols and changes to provide the continuity and the best surgical care for our thoracic cancer patients.

## Disclosure

The authors declare that they have no conflicts of interest.
